# Supplementary material for: Gene Expression Contributes to the Recent Evolution of Host Resistance in a Model Host Parasite System
Source: Front Immunol. 2017 Sep 12;8:1071. doi: 10.3389/fimmu.2017.01071 (PMC5600903; doi:10.3389/fimmu.2017.01071)
Supplement: Supplementary file 1 [file data_sheet_1.pdf]

Lohman et al. 2017 Frontiers in Immunology Supplementary Information

T obtain raw sequence data from UT Austin data archive, from the terminal:

wget http://web.corral.tacc.utexas.edu/Lohman_et_al_2017_FrontiersImmunology/Raw*

To obtain code and data csv files from UT Austin data archive, from the terminal:

wget http://web.corral.tacc.utexas.edu/Lohman_et_al_2017_FrontiersImmunology/Code_Data*

Supplementary Figure 1. Population GO enrichment for Molecular Function (A) and Biological Processes (B).

A)

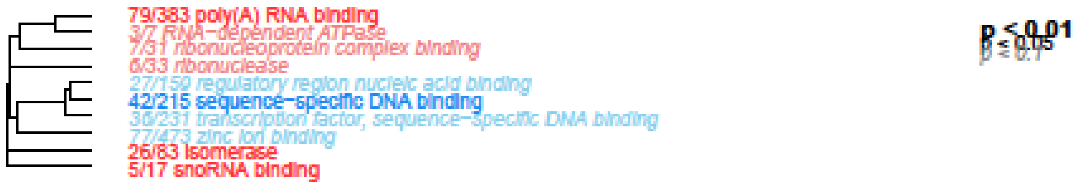

B)

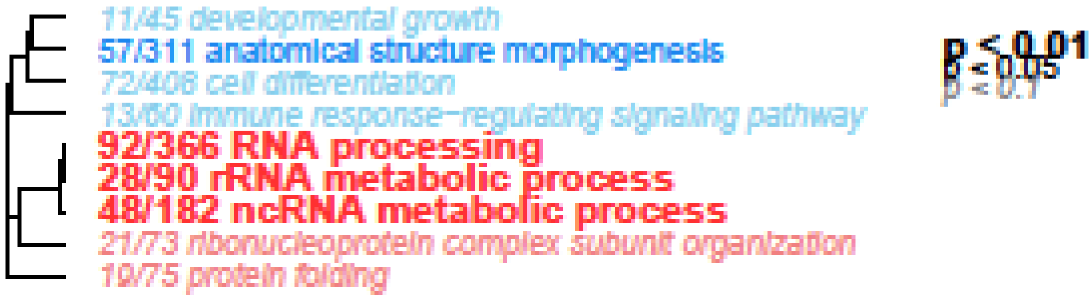

Supplementary Figure 2. Gene-Trait correlations for a signed network with both Rob and Gos fish.

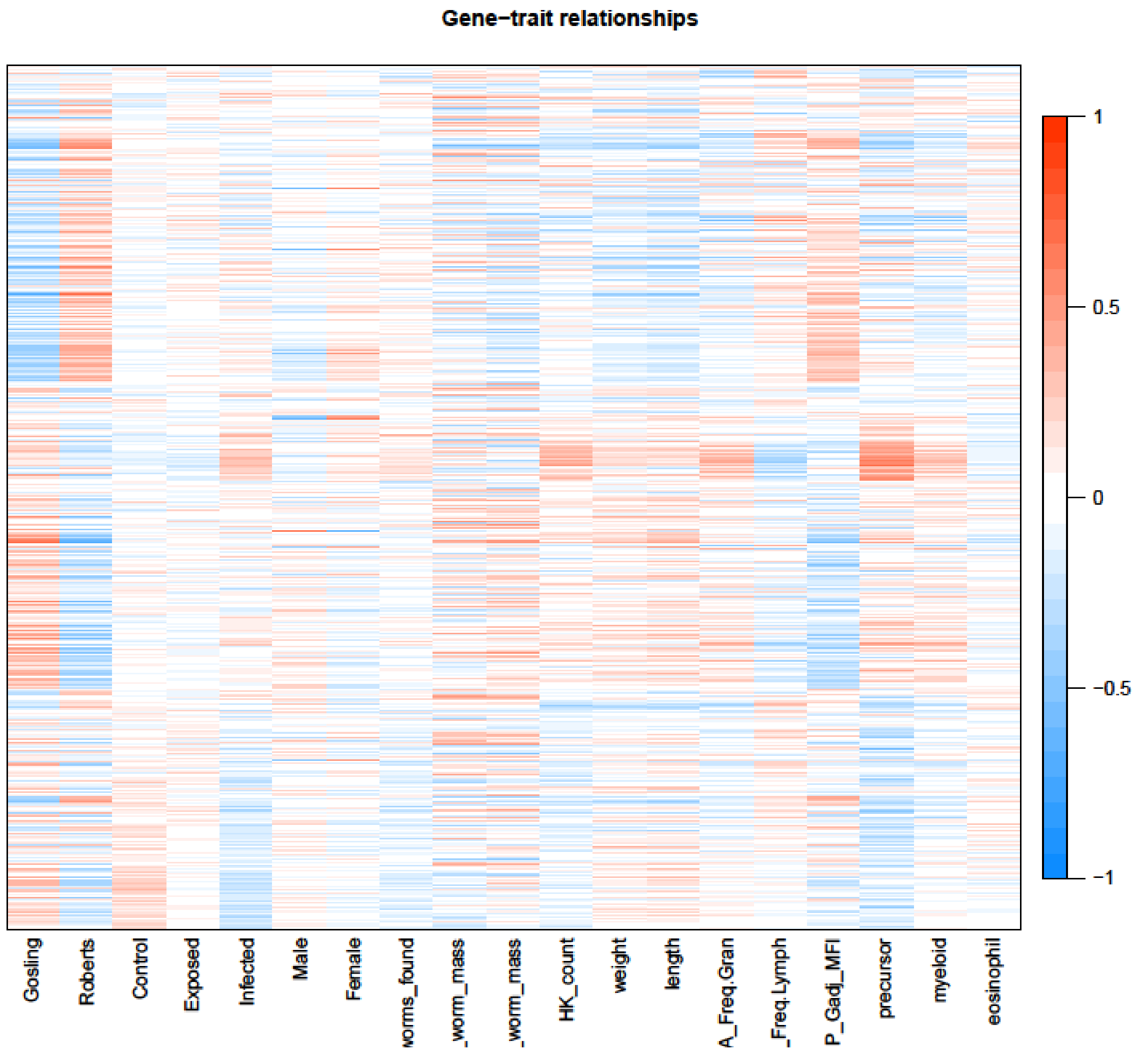

Supplementary Figure 3. Cluster dendrogram from a signed network with both Rob and Gos fish. Colored bars below are groups of genes, or modules.

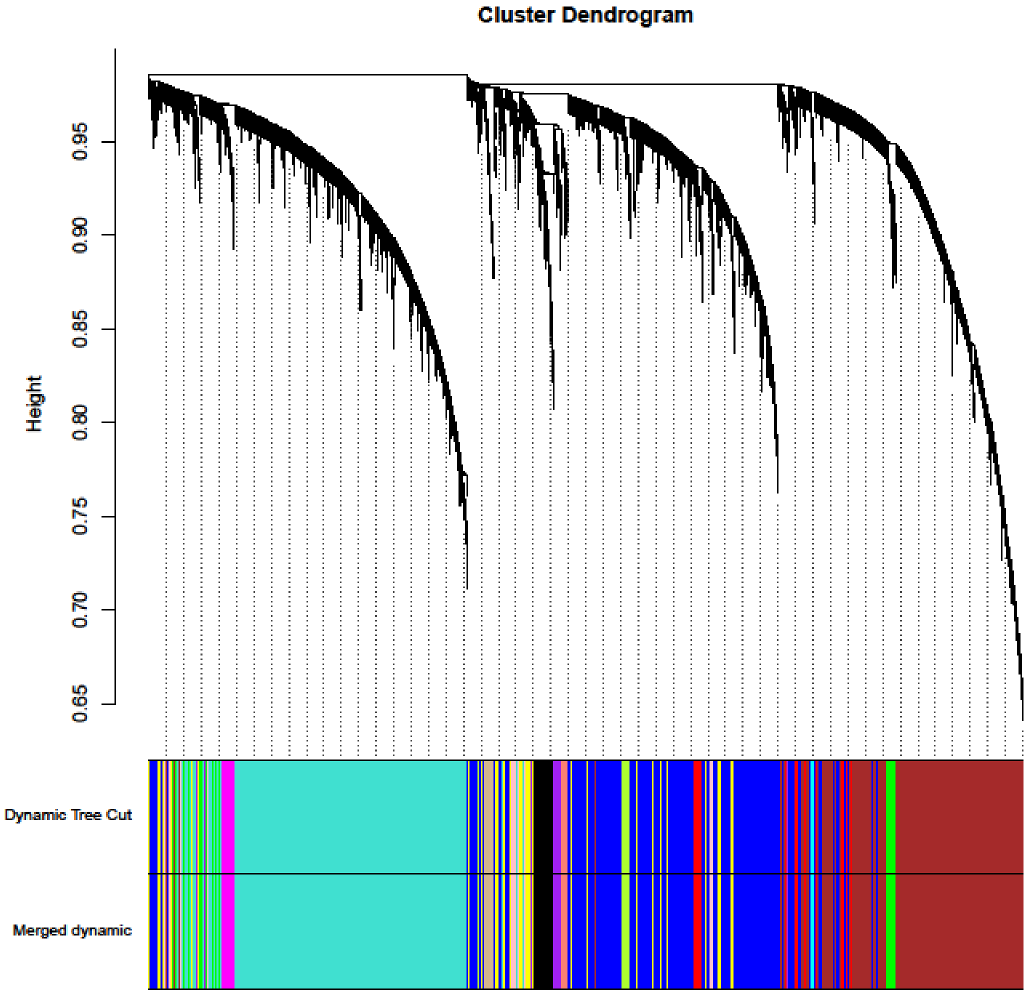

Supplementary Figure 4. Comparison of module membership between signed networks built from Rob only and Gos only fish (see below for additional details on population specific networks). Cell intensity indicates the percent similarity between any two pairs of modules as determined by the fraction of genes shared by both modules.

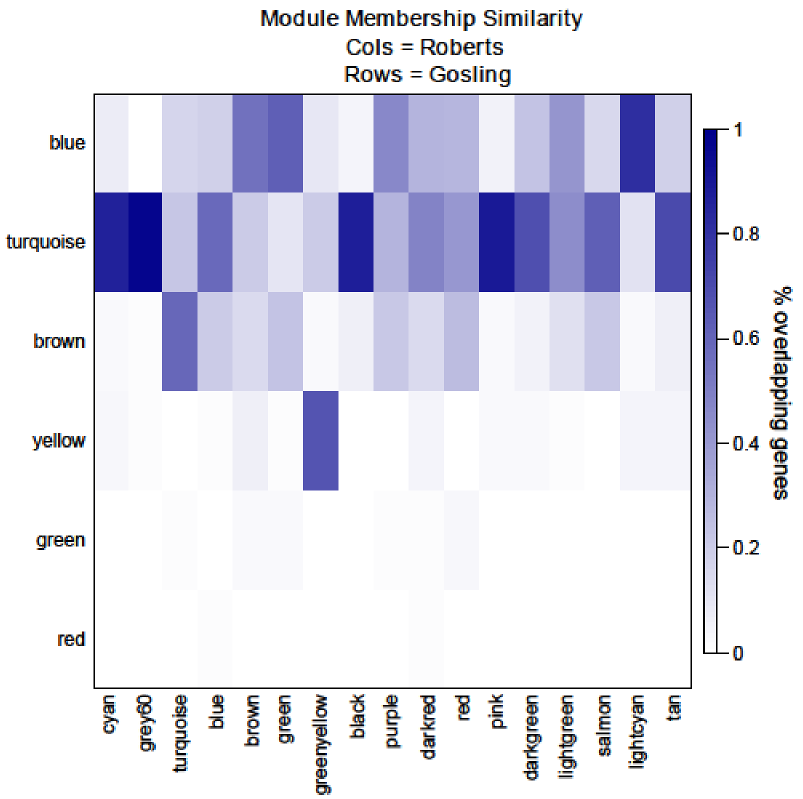

Supplementary Figure 5. Module-trait correlations based on a signed network of only Rob fish. Correlations with p-values greater than 0.1 are omitted.

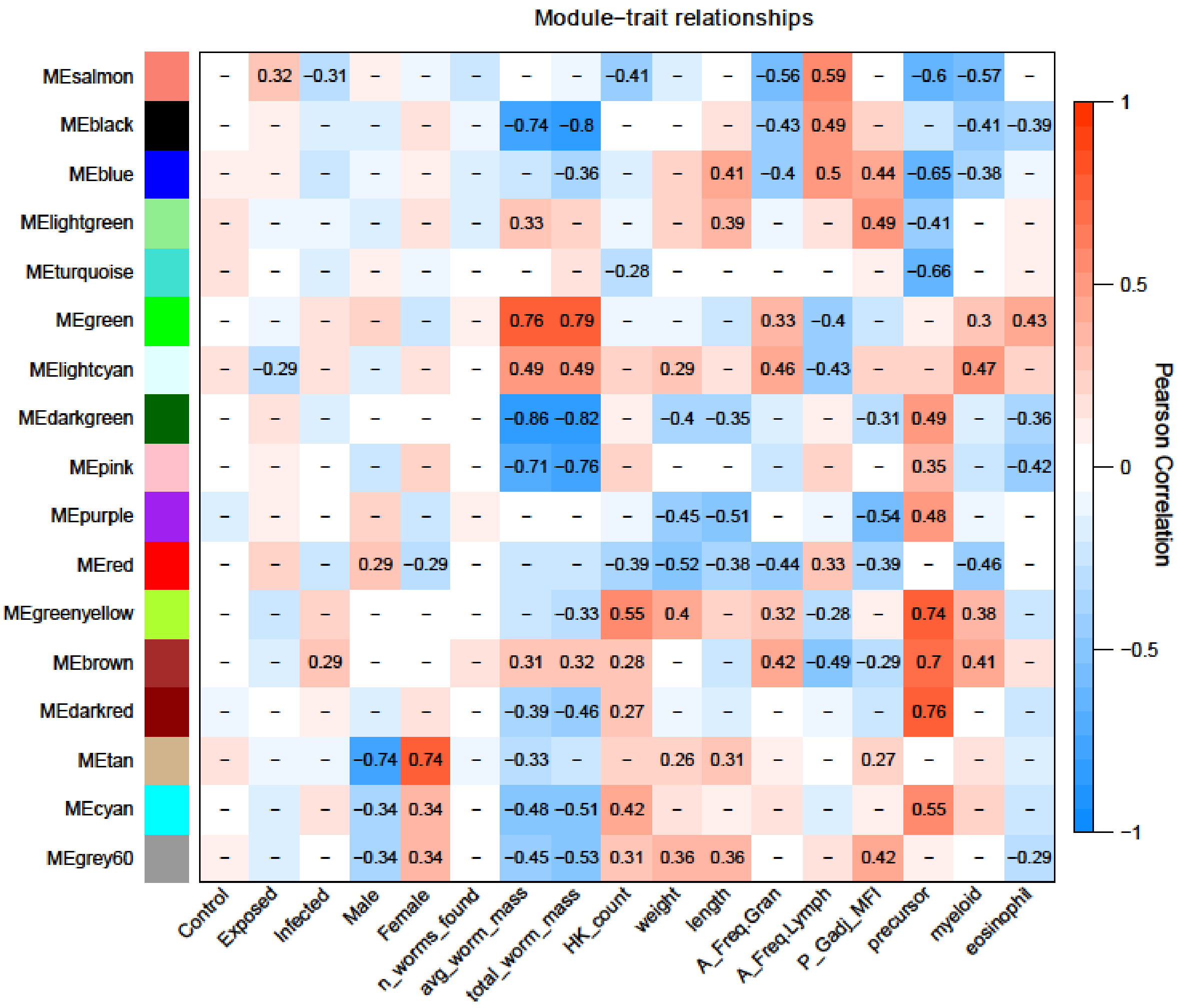

Supplementary Figure 6. Gene-trait correlations based on a signed network of only Rob fish. Each row is 1 gene.

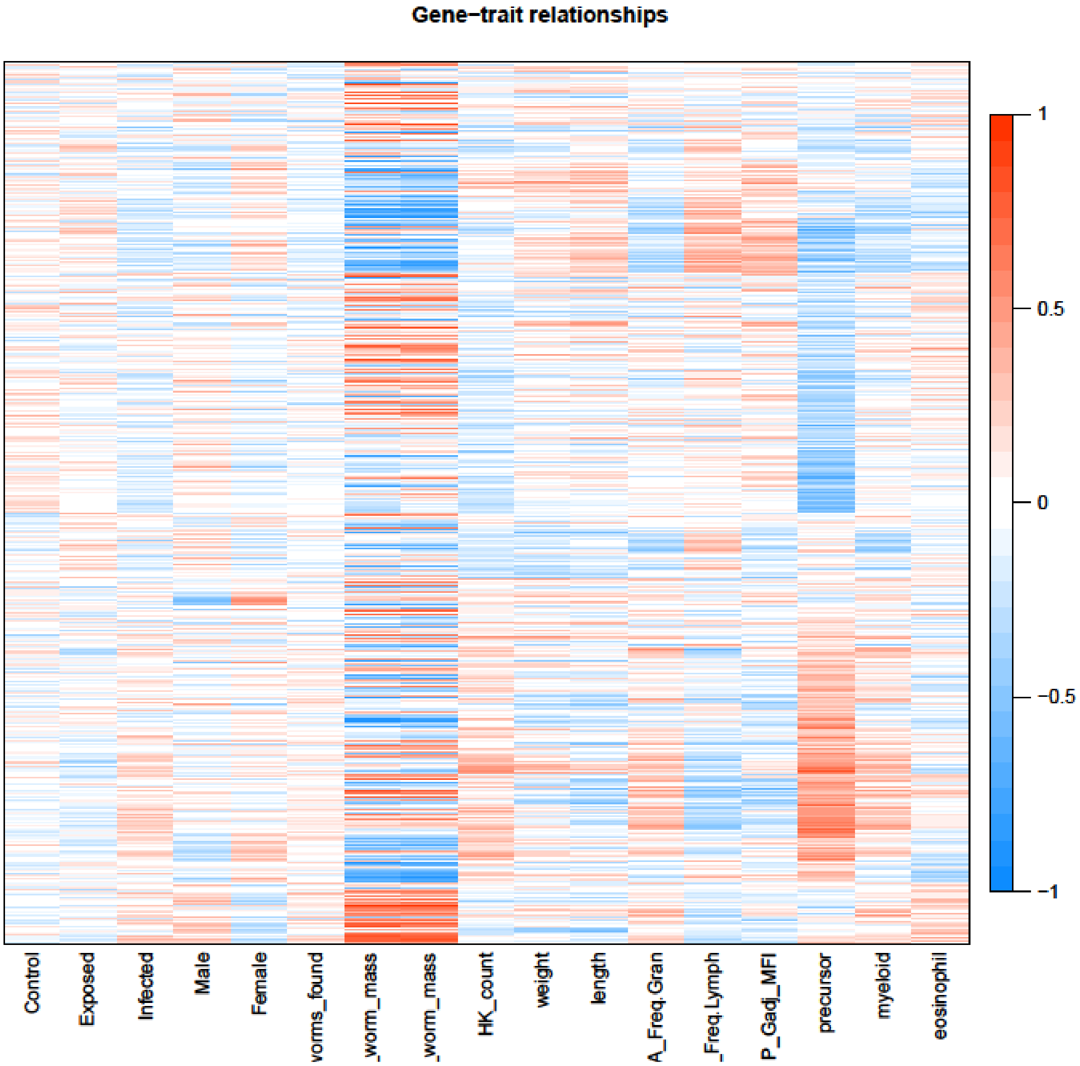

Supplementary Figure 7. Cluster dendrogram for signed network with Rob only fish. Colored bars below are groups of genes, or modules.

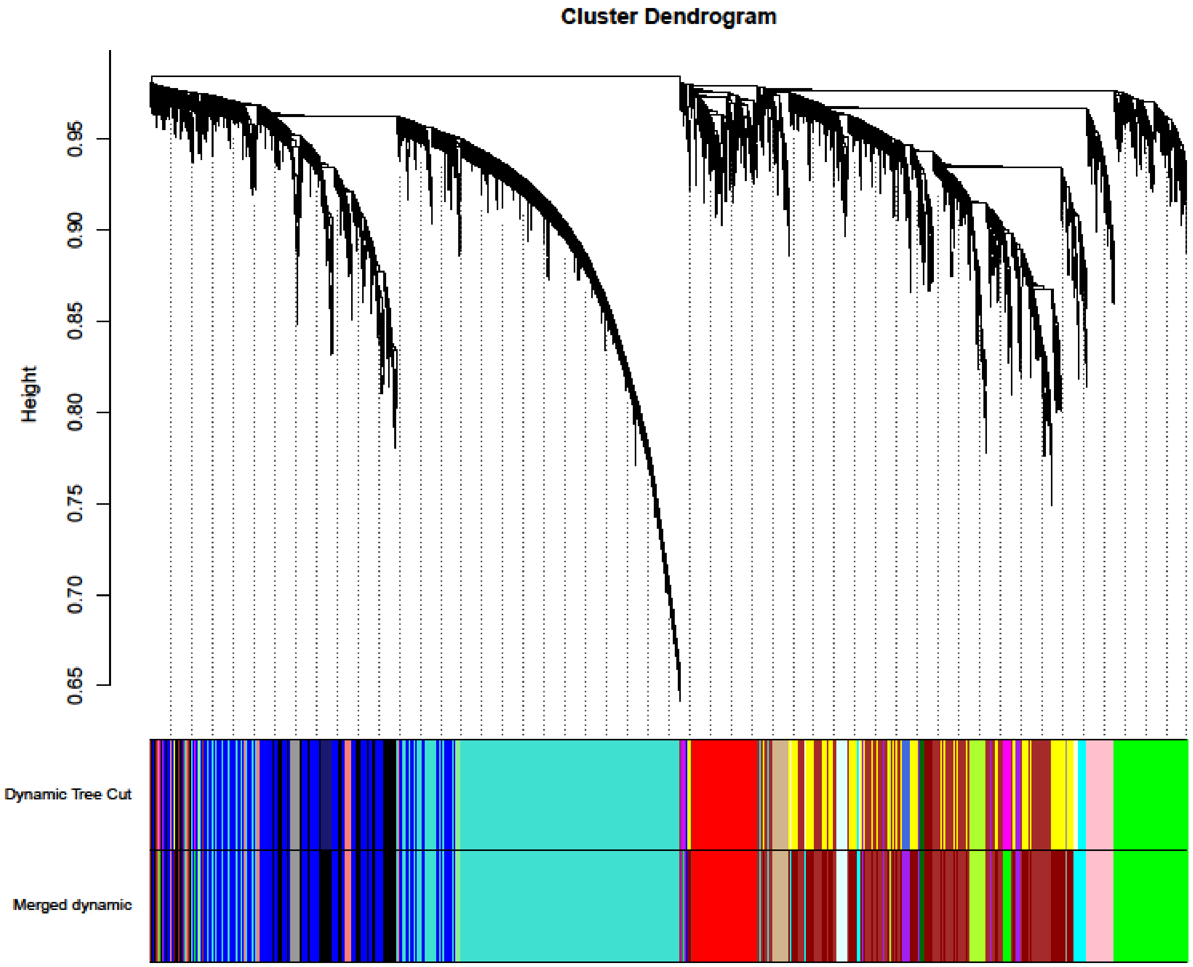

Supplementary Figure 8. Module-trait correlations based on a signed network of only Gos fish. Correlations with p-values greater than 0.1 are omitted.

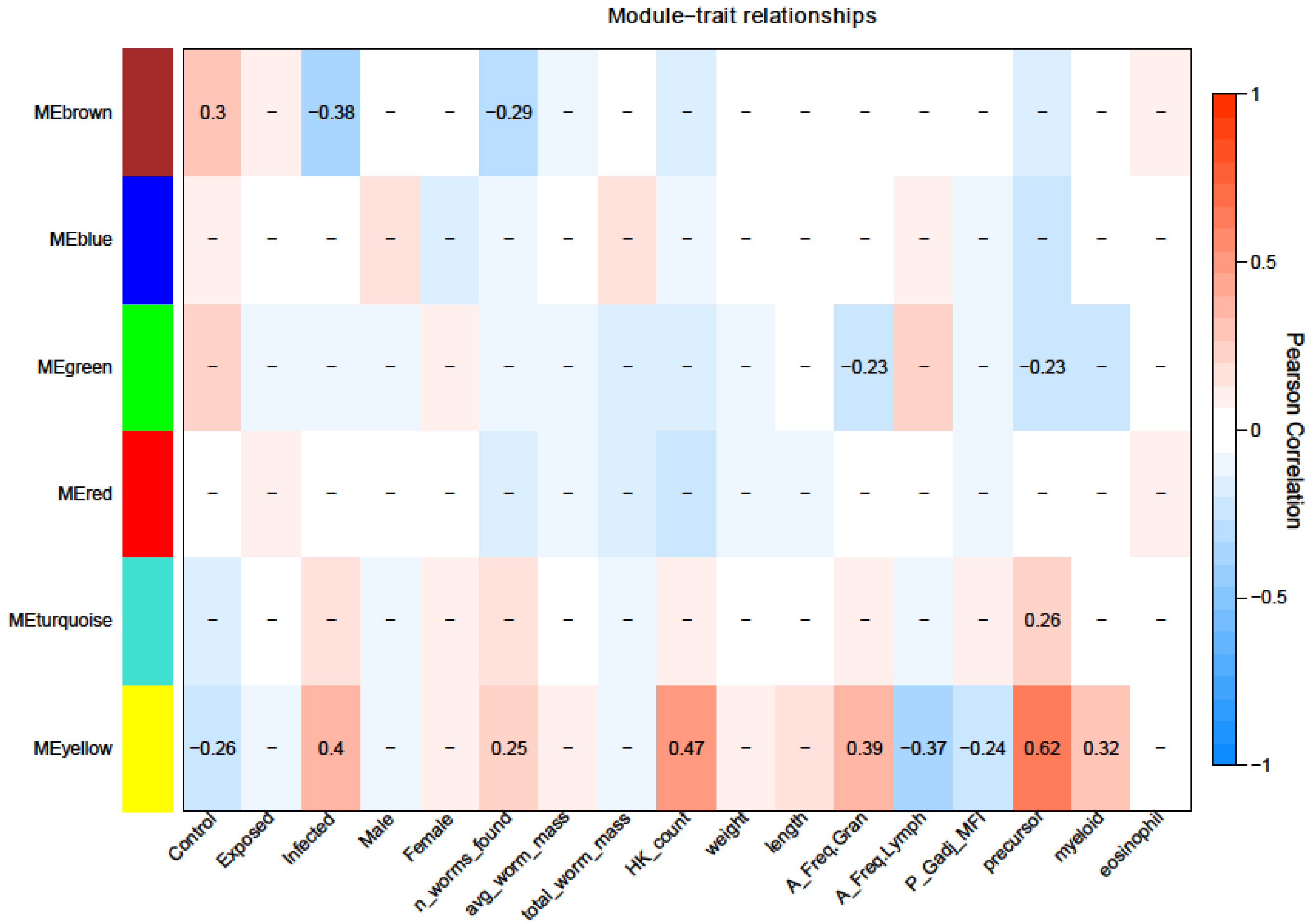

Supplementary Figure 9. Gene-trait correlations based on a signed network of only Gos fish. Each row is 1 gene.

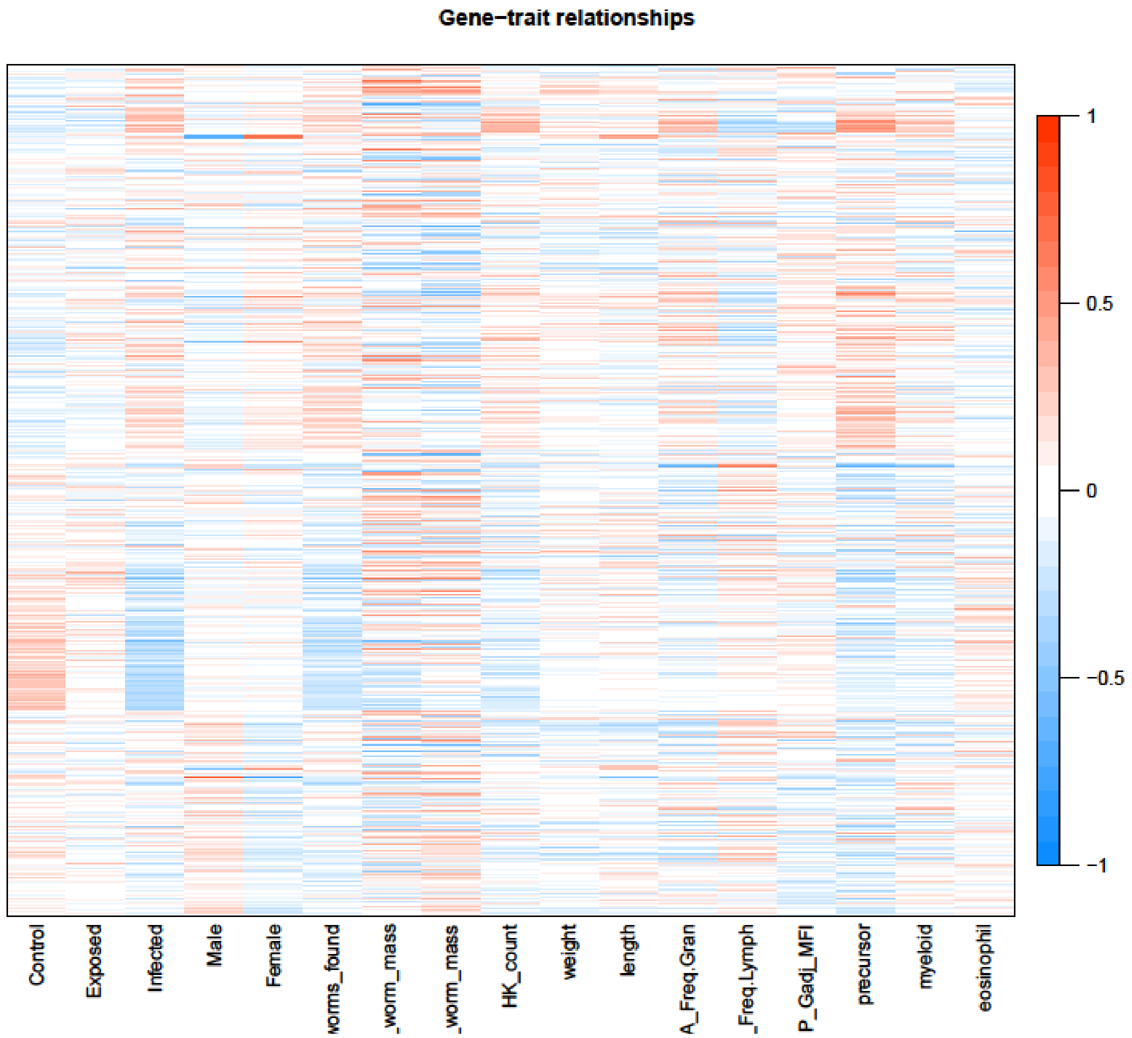

Supplementary Figure 10. Cluster dendrogram for signed network with Gos only fish. Colored bars below are groups of genes, or modules.

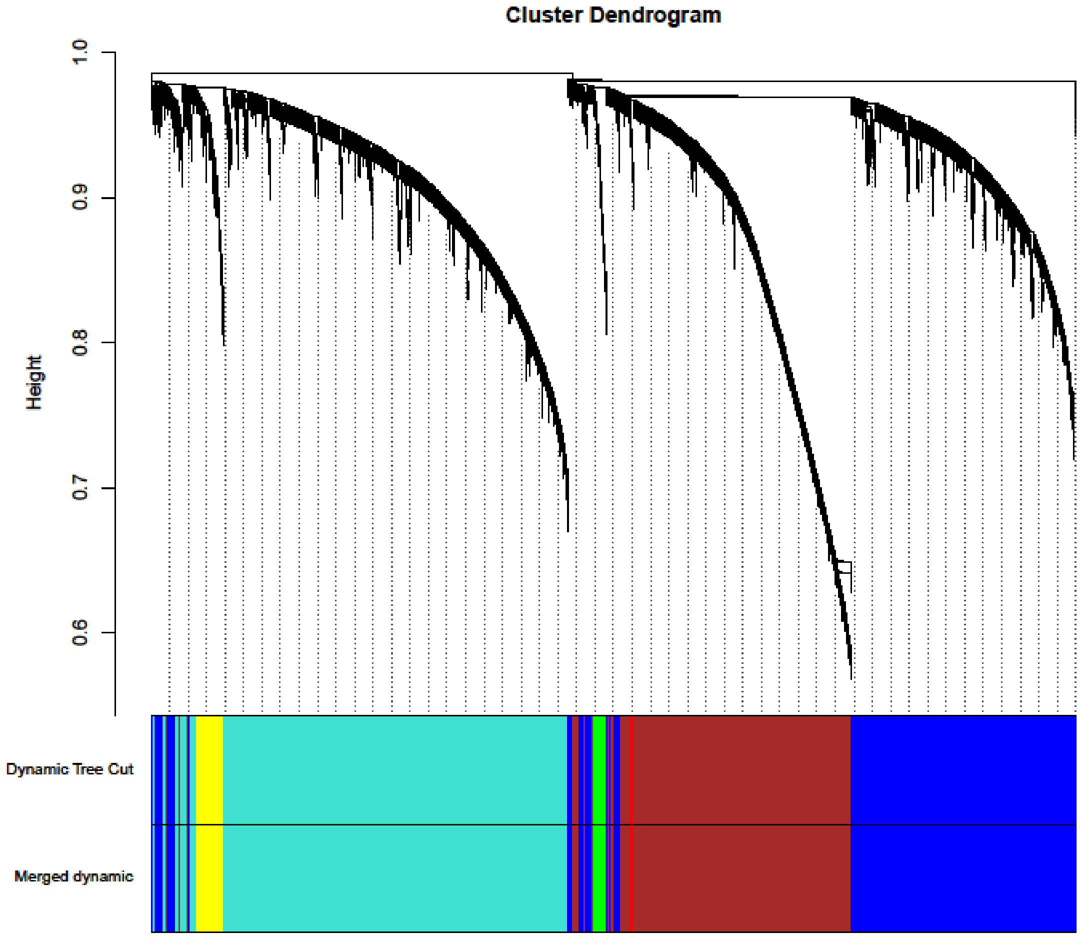

Supplementary Table 1. Family and infection status sample sizes, post outlier removal, for TagSeq libraries. RRXX are Rob families and GGXX are Gos families. The infected fish for GG49 was removed as part of outlier analysis.

Family	Control	Exposed	Infected
**GG12**	2	2	3
**GG13**	2	2	1
**GG15**	1	2	1
**GG16**	1	1	4
**GG17**	1	2	2
**GG22**	2	2	1
**GG32**	2	2	1
**GG42**	2	2	1
**GG47**	2	2	2
**GG49**	2	2	0
**GG8**	2	2	1
**RR10**	2	2	1
**RR12**	2	2	1
**RR13**	2	2	1
**RR15**	2	2	1
**RR18**	2	2	1
**RR30**	2	2	1
**RR44**	2	2	2
**RR52**	2	2	1
